# Raspberry Polyphenolic Extract Regulates Obesogenic Signals in Hepatocytes

**DOI:** 10.3390/molecules23092103

**Published:** 2018-08-21

**Authors:** Bartosz Fotschki, José Moisés Laparra, Michał Sójka

**Affiliations:** 1 Institute of Animal Reproduction and Food Research, Polish Academy of Sciences, 10-748 Olsztyn, Poland; 2 Molecular Immunonutrition Group, Madrid Institute for Advanced Studies in Food (IMDEA-Food), Ctra. de Canto Blanco 8, 28049 Madrid, Spain; moises.laparra@imdea.org; 3 Institute of Food Technology and Analysis, Technical University of Łódź, 90-924 Łódź, Poland; michal.sojka@p.lodz.pl

**Keywords:** raspberry, HepG2, anthocyanins, ellagitannins, high-fat diet, immune-metabolic effects

## Abstract

The aim of this in vitro study was to examine the effect of raspberry polyphenolic extract on the immune-metabolic molecular mechanisms activated by obesity-related signals in hepatocytes (HB-8965^®^). Alterations in endosomal/lysosomal activity (neutral red uptake assay, NR), the expression of selected genes involved with lipid oxidation, and metabolism and inflammation processes in the liver were studied. Hepatocytes were treated with plasma collected from Wistar rats that were fed a high-fat diet (HF), raspberry polyphenolic extract (PP), serine-type protease inhibitors as an agonist of TLR4 (TD) or a combination of PP with HF or TD treatments. The PP added to the experimental treatments modulated hepatic immune-metabolic mechanisms through the upregulation of STAT1, ANGPTL4, and CD44, as well as considerably reducing the NR uptake and downregulation of COX-2 and the multifunctional protein AhR. The kinetic analysis of AhR expression revealed that HF-related molecular mechanisms activated AhR mRNA expression earlier than PP initiated the regulatory effect. In conclusion, PP might be considered a valuable dietary agent that regulates obesity-related signals in hepatocytes. Moreover, taking AhR kinetic behavior into consideration, it can be assumed that PP might modulate the severity of the HF-induced downstream metabolic signaling of AhR.

## 1. Introduction

Lifestyle, including diet and physical activity, constitutes the central axis of global strategies that aim to reduce the severity and pathological manifestations of obesity and its associated comorbidities. Based on dietary strategies, epidemiological data suggest that an increased consumption of polyphenol-rich foods might provide beneficial effects by reducing the occurrence of comorbidities (i.e., type 2 diabetes and cardiovascular diseases) associated with obesity [1,2]. The severity of obesity-related disorders is associated with undesired plasma lipid profile alterations, hepatic metabolic imbalances, innate immune response activation, and metainflammatory processes [3,4]. It has been shown that dietary polyphenols may regulate energy expenditure, fatty acid catabolism in the liver, and antioxidant hepatocellular defenses [5,6]. Diets enriched in polyphenols favor diverse physiological changes; however, there is still little known about the influence of polyphenols on hepatocytes and how these compounds can effectively regulate immune-metabolic events induced by an obesogenic diet.

Most of the recent studies on dietary bioactive compounds and their beneficial effects on the reduction or prevention of obesity-related diseases have mainly focused on green tea catechins, resveratrol, and curcumin [5]. It has been suggested that resveratrol and curcumin attenuate signals from aryl hydrocarbon receptors (AhRs), which play a key role regulating diet-induced obesity [7,8]. Moreover, resveratrol and curcumin might inhibit obesity-related metainflammatory signals driven by toll-like receptor 4 (TLR4) and nucleotide-binding oligomerization domain 2 (NOD2) [9,10,11,12]. Another valuable source of immunonutritional phytochemicals are raspberries. These fruits are known to be a rich source of dietary antioxidants largely due to their high level of ellagitannins and anthocyanins [13,14]. In addition to their robust antioxidant properties, raspberry polyphenols have also been shown to have potential anti-inflammatory effects and the ability to reduce hepatic lipid accumulation [15]. There is limited information about how raspberry polyphenols might regulate molecular pathways involved in anti-obesity effects. Viladomiu et al. [16] suggested that ellagitannins and anthocyanins might regulate the inflammatory processes through the downregulation of cyclooxygenase 2 (COX2) expression. Additionally, a nutritional study performed on rats with metabolic syndrome symptoms induced by a high-fat diet showed that ellagic acid attenuates diet-induced impairment of glucose tolerance and reduces NF-κB protein levels in the liver [17]. In addition, our previous nutritional experiments with dietary extracts from raspberries have indicated that these extracts are effective at decreasing total triglycerides and cholesterol in the blood and modulate hepatic lipid metabolism in rats fed with a high-fat diet [18,19,20]. The regulatory effects observed in the liver partially support the hypothesis that raspberry extracts can reduce liver disorders induced by a high-fat diet. However, the underlying molecular mechanisms of these beneficial effects remain poorly understood. 

In view of the aforementioned information, the objective of the present study was to investigate the impact of a raspberry polyphenolic extract on immune-metabolic biomarkers in hepatocytes and compare the impact of this extract with changes caused by plasma obtained from animals fed a high-fat diet.

## 2. Results

### 2.1. Characterization of the Raspberry Polyphenolic Extract and Plasma Samples

The raspberry polyphenolic extract from juice (PP) used in the study contained 15.7 g/100 g phenolic compounds, most of which were identified as ellagitannins (8.4 g/100 g) and anthocyanins (6.3 g/100 g) (Table 1). 

**Figure 1:**
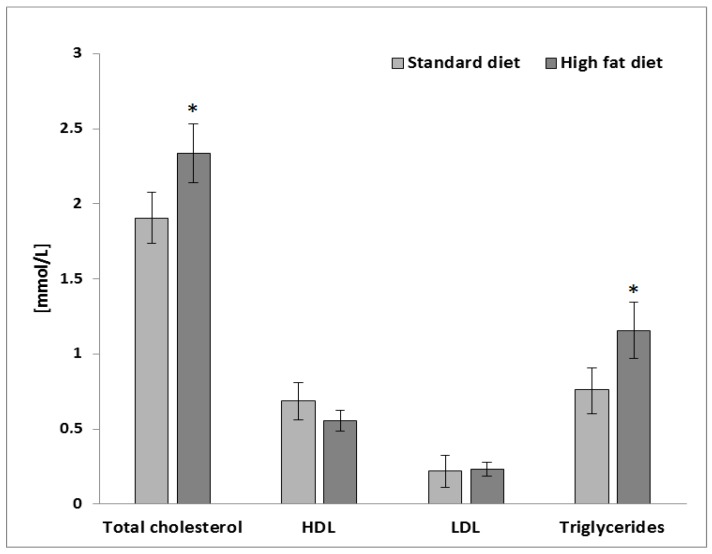
Plasma lipid profile from Wistar rats fed with a standard diet and high-fat diet. Values are the mean ± SEM (*n* = 8). The mean values within a symbol (*) were significantly different according to a Student’s *t* test (*p* ≤ 0.05).

To induce obesity-related changes in the hepatocytes (HepG2 cells), plasma samples obtained from a preclinical study using Wistar rats fed with a high-fat diet were used [18]. The plasma samples from an in vivo model of a high-fat diet contained significantly higher values of the total cholesterol (TC) and triglycerides (TG) in comparison to the animals fed with a standard diet for laboratory rodents (*p* ≤ 0.05; Figure 1). 

### 2.2. Activity of the Endo/Lysosomal Compartment

**Figure 2:**
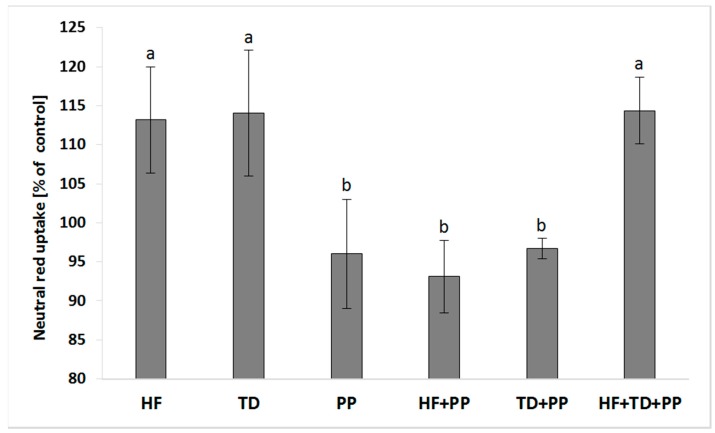
Neutral red uptake in cell cultures of hepatoblastoma (HB-8065^®^) cells exposed to plasma collected from Wistar rats fed with a high-fat diet (HF), raspberry extract (PP), HF combined with raspberry extract (HF + PP), and serine-type protease inhibitors obtained from *Triticum durum* (TD) and TD combined with raspberry extract (TD + PP). Values are the mean ± SD (*n* = 4). The mean values within a superscript letter (a or b) were significantly different by Duncan’s post hoc test (*p* ≤ 0.05).

We investigated whether cellular responses affecting the endosomal formation/trafficking promoted by plasma samples might be regulated by PP (Figure 2). The concurrent exposure of HepG2 cells to the PP and either a high-fat diet or the extract from *Triticum durum* (TD) significantly decreased the activity of the endo/lysosomal compartment in relation to the independent challenge of plasma samples or the extract from *Triticum durum*. The potential aggravation of cellular effects caused by plasma samples was approached by mixing a protein extract obtained from *Triticum durum* as a potent immune-metabolic modulator of cellular responses. The PP was unable to counteract the increased induction mediated by the HF/TD mixture on the endo/lysosomal activity. 

### 2.3. mRNA Expression of Selected Biomarkers

**Figure 3:**
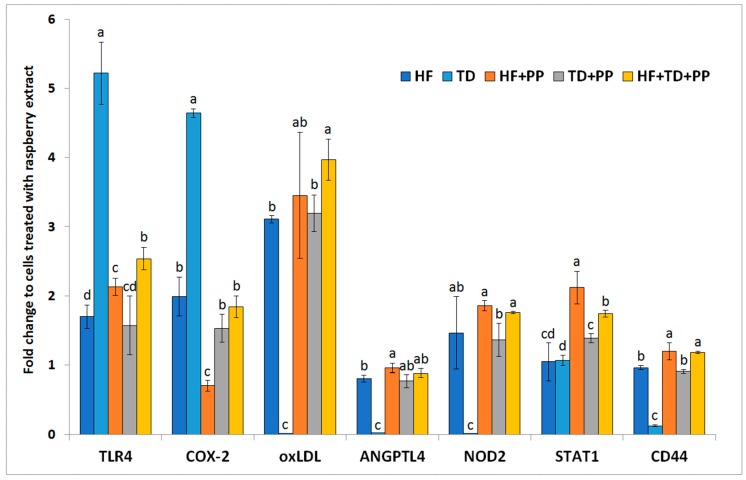
mRNA expression of selected biomarkers in hepatocytes (HB-8965^®^) after exposure (3 h) to plasma collected from Wistar rats fed with a high-fat diet. TLR4, toll-like receptor 4; COX-2, cyclooxygenase-2; oxLDL, oxidized low-density lipoprotein receptor; ANGPTL4, angiopoietin-like 4; NOD2 nucleotide-binding oligomerization domain 2; STAT1, signal transducer and activator of transcription 1; CD44, cluster of differentiation 44. Samples from Wistar rats were mixed with: (1) the raspberry extract (HF + PP); (2) serine-type protease inhibitors obtained from *Triticum durum* (TD); and (3) joint addition of combination of the HF, TD, and PP (HF + TD + PP). Values are the mean ± SD (*n* = 3). Values with a different superscript letter (a, b, c, d) were significantly different by Duncan’s post hoc test (*p* ≤ 0.05).

According to the expression profiles of the selected biomarkers (Figure 3), we identified different immune-metabolic cellular responses of hepatocytes when exposed to independent or concurrent exposures of the different samples with PP. The TD-induced upregulated expression of TLR4 was counteracted by PP in cultures challenged by the concurrent addition of those samples. However, PP was unable to inhibit the HF-induced increase in TLR4 expression. The higher activation of the TLR4 signaling pathway might be partially related to the upregulation of COX-2 expression. The COX-2 regulates the synthesis of prostaglandins from arachidonic acid and polyunsaturated fatty acids, and, thereby, influences the severity of pathological responses [21]. In this study, it was observed that PP added to the different treatments significantly (*p* ≤ 0.05) reduced the expression of COX-2 in the hepatocytes (65% for HF, 68% for TD). These differences might be partially associated with activation of the other molecular mechanisms modulating activation of ANGPTL4 and/or oxLDL receptors. ANGPTL4 regulates activation of the lipoprotein lipase, and thus fat partitioning and liver fat absorption [22]. In concordance with this, the addition of PP to the different treatments caused considerable (*p* < 0.05) upregulation of ANGPTL4 transcription levels (~17% for HF and ~30 times for TD). Only the concurrent exposure of cells to PP and TD elevated (*p* < 0.01) the expression level of oxLDL receptors, known as the “scavenger receptors” with important functions that modulate immune-metabolic responses of hepatocytes [8]. The aforementioned changes in biomarkers associated with lipid homeostasis were linked to changes, at significantly different rates, of the transcripts from STAT1 (an important regulatory effector of energy expenditure and fat mobilization) in the presence of PP (~101% for HF, ~30% for TD and ~63% for HF + TD). The potential immunomodulatory effect of PP affected the expression of innate immune receptors such as CD44 (~20% for HF and six times for TD) and NOD2 (~100 times). The observed regulatory effects of raspberry polyphenolic extract suggested that polyphenols may help induce metabolic responses to promote adequate immune responses that prevent liver injury under obesity-induced stress. 

### 2.4. Modulated Expression of the AhR in Response to PP Exposure

**Figure 4:**
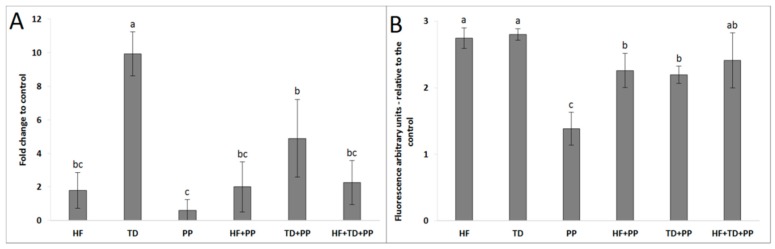
Expression of mRNA (**A**) and ligand protein (**B**) of aryl hydrocarbon receptor (AhR) in cell cultures of hepatoblastoma (HB-8065^®^) exposed for 3 h to plasma collected from Wistar rats fed with a high-fat diet (HF), raspberry extract (PP), HF combined with raspberry extract (HF + PP), and serine-type protease inhibitors obtained from *Triticum durum* (TD) and TD combined with raspberry extract (TD + PP). Values are the mean ± SD (*n* = 3). The mean values with a different superscript letter (a, b, c) were significantly different by Duncan’s post hoc test (*p* ≤ 0.05).

**Figure 5:**
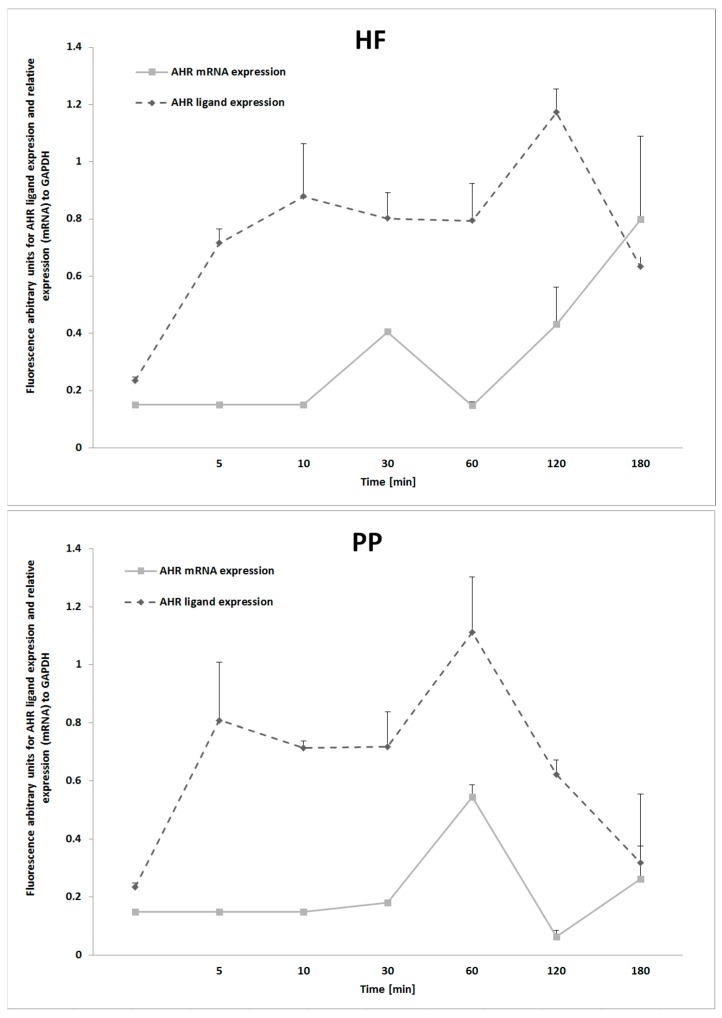
Kinetic analysis of aryl hydrocarbon ligand (AhR) protein and mRNA expression in cell cultures of hepatoblastoma (HB-8065^®^) cells exposed to plasma collected from Wistar rats fed with a high-fat diet (HF) and raspberry extract (PP). Values are the mean ± SD (*n* = 3).

Our study deepened the understanding of the aryl hydrocarbon receptor, a multifunctional signal protein linking metabolic alterations with imbalances in immune responses. It was observed that hepatocytes, after treatment with HF and TD, considerably upregulated (*p* ≤ 0.01) ligand expression of the AhR (Figure 4). As expected, because AhR participates in the signaling cascade of innate immune “Toll-like” receptors, the TD treatment upregulated AhR expression at the mRNA level. Notably, the AhR level was significantly reduced (*p* ≤ 0.05) when PP was added to the medium (~18% for HF, ~22% for TD). According to the results, it might be assumed that the immune-metabolic effect of the PP was partially related to AhR activity in hepatocytes. Further kinetic analysis revealed that HF treatment and PP activated expression of AhR with different patterns (Figure 5). The HF started upregulating the AhR (mRNA) after 30 min, and at 180 min, the mean value of the expression level was increased to approximately 99%. However, cell cultures exposed to PP presented a maximum AhR expression at 60 min, and after 180 min, approximately 48% of the expression level decreased. The results from AhR ligand expression presented the highest protein level after 120 min exposition to HF treatment and 60 min exposition to PP treatment. Taking into consideration the AhR kinetic behavior, it can be hypothesized that feeding PP could modulate the severity of HF-induced metabolic downstream signaling of AhR.

## 3. Discussion

This study showed that administration of PP extract could represent an effective approach to control the hepatic immunometabolic effects associated with a high-fat diet. Taking advantage of the known participation of hepatocyte toll-like receptors (TLRs) on obesity-induced hepatic metabolic alterations, inflammation, and insulin resistance [10,23], the studies also extended this knowledge.

Polyphenols (e.g., ellagitannins and anthocyanins) are poorly absorbed in their native physicochemical form in the gastrointestinal tract [18,24]. Estimated values for polyphenol bioavailability are commonly found at approximately 5% [25,26]. Thus, in this study we prepared different mixtures containing PP concentrations at 5% to approach their influence on the expression levels of selected biomarkers in hepatocytes (HepG2 cells). Exposure of HepG2 cells to 5% of the raspberry polyphenolic extract was able to significantly reduce neutral red uptake. This suggested that even low concentrations of polyphenols within the bloodstream could be responsible for certain bioactivity affecting absorption or vesicle trafficking processes. Previous studies established the positive effect of polyphenols increasing energy expenditure through upregulating mitochondrial uncoupled proteins [5]. This dissipates the mitochondrial proton gradient causing energy depletion, and thereby disruption of numerous cellular processes, including vesicular trafficking. Thus, raspberry polyphenolic extract could interfere with trafficked lipoproteins into the lysosomes, implying limited, if any, recycling back to the cell surface (i.e., innate immune receptors). Accordingly, feeding animals with a high-fat diet containing 7% standard and a finely ground seedless fraction of raspberry pomace significantly reduced the hepatic concentration of cholesterol [18]. Because of the high rate of hepatocellular membrane turnover, polyphenol-mediated control of endocytic vesicles could probably make a larger contribution to anti-obesogenic effects than previously recognized. However, limited studies have been conducted along these lines and several of them showed contrasting anti-obesogenic impacts of dietary polyphenols, likely due to the distinct experimental conditions [26,27].

It has been reported that plant-derived polyphenols (i.e., ellagic acid, caffeic acid, coumaric acid, ferulic acid, quercetin, rutin, kaempferol, and catechins) [28] could have a dual role exerting both pro- and antioxidant properties [29]. As an aspect of the balance between such functions, it affected, among others, their metal-reducing and chelating potential as well as pH. In this study, proinflammatory signals driven by the pro-obesogenic TLR4 are controlled by raspberry polyphenolic extracts rich in ellagic acid conjugates (Figure 3). It could be hypothesized to occur through limiting the iron-mediated selective modulation of TLR4-activated inflammatory responses [30]. This effect is in accordance with the lower transcript level quantified for COX-2, which mediates the rate-limiting step in arachidonic acid metabolism [31]. In addition, these results seemed to conflict with in vivo data in which raspberry pomace extracts (obtained by standard grinding) upregulate defined members of the endocrine fibrosis growth factor family (i.e., FGFR4 and FGF19) [18]. It has been shown that the metabolic response to FGF19 signaling in the liver is impaired in obese patients with nonalcoholic fatty liver disease (NAFLD) (steatosis) and steatohepatitis. Additionally, there was a straight association between FGFR4/FGF19 and beta-catenin target gene (*cyclin D1*, *CD44*, *c-jun*, *COX-2*, and *UPAR*) expression levels [32]. Here, it must be kept in mind that the endocrine fibroblast growth factor (FGF)family may play a dual role and exert protective metabolic effects limiting lipotoxicity by promoting hepatic fatty acid activation [33,34]. The latter would imply the need for modifying hepatic surrounding events, which could be expected to be mediated by polyphenols. Taken together, polyphenols could prevent the activation of p38 and JNK by either: (i) selectively modulating TLR4-driven inflammatory responses towards the TRIF adaptor molecule [30], or (ii) by arachidonic acid [35]. More controlled obesogenic processes could be expected at the liver level. Accordingly, animals fed a high-fat diet supplemented with a raspberry extract exhibited lower mean values (by 4.9%) for liver mass in relation to body weight [18].

Based on the expression (mRNA) profiles of ANGPTL4 and the oxLDL receptor, we can gain insights about the potential impact of raspberry polyphenolic extract in lipid homeostasis. A high-fat diet has been found to decrease ANGPTL4 in blood plasma [36]. In this study, raspberry extract was able to abolish the negative high-fat diet-mediated modulation of ANGPTL4 and upregulate its expression, which would be translated to inhibited intestinal lipid digestion [37]. However, raspberry polyphenolic extract does not appear to exert significant effects on the high-fat diet-related modulation of oxLDL receptor expression, which is known to bind lipoproteins with a mechanism dependent upon fatty acid binding [38]. The latter could be supported, at least in part, by the significant effect of the administration of a 0.30% (*w*/*w*) raspberry extract diet that reduced (by 33.4%) rat plasma TGs [19].

An overview of in vivo studies revealed the convergence of distinct innate and metabolic signals in the development of obesity and its associated comorbidities. As shown, raspberry polyphenolic extract increased NOD2 transcription in TD samples or plasma samples from animals fed an HF. Obesity has been associated with increased expression levels of NOD2 in hepatocytes [11]; however, the exact role of NOD2 in obesity remains unknown. The experimental design of this study did not allow final conclusions to be made about NOD2-TLR4 interactions. Nevertheless, an inverse relationship was observed between mRNA expression levels in the presence of the raspberry polyphenolic extract. The inhibitory interaction of the examined extract in TLR4 transcripts can decrease the glycolytic activity as a major physiological consequence. A critical overview of the literature revealed STAT1 as a rate-limiting “sensor” orchestrating regulatory signals for energy expenditures. These effects could help to explain, at least in part, the weight loss observed in animal models fed food polyphenols [30].

To further study the influence of raspberry polyphenolic extracts on regulatory signals affecting adipose tissue inflammation in obesity and hepatic leukocyte recruitment, the expression levels of CD44 were monitored. Either extracts or plasma samples from animals fed an HF increased the transcriptions of CD44 to a similar extent. The concurrent exposure of raspberry polyphenolic extracts and plasma samples from animals fed a high-fat diet increased the expression levels of CD44 by 20% in relation to the effect caused by the independent addition of raspberry polyphenolic extracts. Recent literature reports have suggested the development of biased effector memory CD4 T cell differentiation characterized by increased CD44 under obesity-induced metabolic stress [39]. This immune response promotes the development of Foxp3-T (Tr1) cells, which exhibit a marked potential (higher than Foxp3+) to inhibit the secretion of IL-1β [40]. Moreover, CD44 constitutes an effective activator of the macrophage inhibitory factor-induced p38 activation in injured liver [41]. Together, these results may suggest that PP enhances the usage of CD44 to develop immune-metabolic responses to preserve liver function in obesity [41].

Although the convergence between metabolic and immune stimuli is documented in the literature, there is compelling in vivo evidence regarding the contrasting effects of dietary polyphenols in weight loss and obesity-associated comorbidities. Thus, the effects of the different samples on the expression of AhR, as a key player in diet-induced adiposity and metabolic disorders influencing energy expenditure, were studied [42]. All assayed samples caused a “double peaked” time-course profile in the cytoplasmic levels of AhR. The fold change in luminescence for the first peak was similar in cultures exposed to raspberry polyphenolic extracts and in samples from animals that were fed a HF. The differences in the “second peak” appeared sharper and clearer. Here, the data showed variations in the luminescence that, taken together, may suggest that discrepancies in the different sensitivities of signaling via AhR could determine the observed physiological effects. From a nutritional point of view, these effects could be viewed as a need to adapt dietary recommendations in order to consume polyphenols independently and prior to dietary intake of fat-containing foods. 

## 4. Materials and Methods 

### 4.1. Preparation of the Raspberry Polyphenolic Extract

Raspberry juice, obtained as described in Supplementary Materials 1, containing 11% dry substance (as Brix) served as the raw material for the preparation of the raspberry phenolic extract. The juice was absorbed on an 8 L Amberlite XAD absorption bed placed in a 10 L column. Prior to processing, the sorbent was conditioned as recommended by the manufacturer. In total, 48 L of juice (corresponding to 6 BVs (bed volumes)) were passed through the column, at a flow rate 1 BV/h. Next, the column was washed with 2 BV of water and 1 BV of 5% ethanol. The desorption was carried out by washing the column in counter-current with 1.5 BV of 25% ethanol. The eluate (total volume 12 L) was concentrated with a rotary evaporator in 2 steps: first, to remove ethanol and second to reduce the total volume to 1 L. Concentrated extract containing 14.6% dry matter (as Brix) was frozen and freeze-dried.

### 4.2. Quantification of the Polyphenols

The concentrations of ellagitannins, ellagic acid, anthocyanins, and flavonols were determined in extracts diluted with methanol (1 mg/mL) using a Knauer Smartline HPLC system with a photodiode array detector (Berlin, Germany) coupled with a Gemini C18 column (110 Å, 250 × 4.60 mm; 5 μm, Phenomenex, Torrance, CA, USA). Phase A was 0.05% phosphoric acid in water, phase B was 0.05% phosphoric acid in 80% acetonitrile, the flow rate was 1.25 mL/min, the sample volume was 20 μL, and the temperature was 35 °C. The following gradient was applied: stabilization for 5 min with 4% phase B, 4–15% B for 5–12.5 min, 15–40% B for 12.5–42.5 min, 40–50% B for 42.5–51.8 min, 50–55% B for 51.8–53.4 min, and 4% B for 53.4–55 min. The following standards were used for the identification of the polyphenols: ellagic acid, flavonols (quercetin-3-*O*-glucoside, kaempferol-3-*O*-glucoside, quercetin, kaempferol, tiliroside), pelargonidin-3-*O*-glucoside, p-coumaroic acid, and ellagitannins (hexahydroxydiphenoyl-d-glucose and agrimoniin). The absorbance was measured at 280 nm (*p*-coumaroic acid, tiliroside, hexahydroxydiphenoyl-d-glucose, and agrimoniin), 360 nm (ellagic acid, quercetin, kaempferol, and kaempferol glycosides) and 520 nm (anthocyanins). More details about separation and detection conditions are described elsewhere [43].

The concentration of proanthocyanidins in the extracts was determined by HPLC following their breakdown in an acidic environment with excess phloroglucinol, according to Kennedy and Jones [44]. The obtained products were separated using a Knauer Smartline chromatographic system (Berlin, Germany) equipped with a UV–Vis detector (PDA 280, Knauer, Berlin, Germany) and a fluorescence detector (Shimadzu RF-10AXL, Kyoto, Japan) coupled with a Gemini C18 column (110 Å, 250 × 4.60 mm; 5 μm, Phenomenex, Torrance, CA, USA). The separation conditions were as described by Kosmala et al. [45]. Identification was performed at 280 nm using a UV–Vis detector.

Ellagitannins were confirmed by HPLC-ESI-MS using a Dionex UltiMate 3000 UHPLC and a Thermo Scientific Q Exactive series quadrupole ion trap mass spectrometer [20]. A Phenomenex Luna 5 μm C18 (250 × 4.6 mm) column was used with a binary gradient of 1% formic acid as mobile phase A and water/acetonitrile (1:4 *v*/*v*) as mobile phase B at a flow rate of 1.0 mL/min. The gradient program was 5% of mobile phase B for the first 6.5 min, 5–15% B from 6.5 to 12.5 min, 15–45% B from 12.5 to 44 min, and 45–75% B from 44 to 45 min. Isocratic conditions were at 75% B from 45 to 50 min and 75–5% B from 50 to 52 min, and column equilibration was performed at 5% B from 52 to 65 min. The mass spectrometry analysis was performed in a negative ion mode under the following conditions: capillary voltage, +4 kV; sheath gas pressure, 75 au (arbitrary units); auxiliary gas, 17 au; and scan range, 200 to 2000 *m*/*z*.

The equipment used for the identification of anthocyanins was the same as that described for identification of ellagitannins. The solvents used for separations were as follows: solvent A: 1% (*v*/*v*) formic acid in water and solvent B: 1% (*v*/*v*) formic acid in methanol. The following gradient was used: 0−30 min, 20−65% (*v*/*v*) B; 30−31 min, 65−100% (*v*/*v*) B; 31−33 min, 100% (*v*/*v*) B; 33−34 min, 100−20% (*v*/*v*) B; and 34−45 min, 20% (*v*/*v*) B. A column (150 mm × 4.6 mm i.d., 3 μm, Gemini−NX C18 110 Å) was used with a Gemini-NX C18, 4 mm × 3 mm i.d. precolumn (Phenomenex, Torrance, CA, USA). The mass spectrometry analysis was performed in a positive ion mode under the following conditions: ion-spray voltage of 3.8 kV; sheath gas pressure, 60 au (arbitrary units); auxiliary gas, 20 au; scan range, 250 to 1000 *m*/*z*. More details about separation and detection conditions are described elsewhere [46].

### 4.3. Extraction of the Salt-Soluble Protein Fraction

Samples of *Triticum durum* were finely milled and homogeneously ground. The obtained flour was extracted with a buffered saline solution (PBS, 137.0 mM NaCl, 2.7 mM KCl, 8.1 mM Na_2_HPO_4_, 1.5 mM KH_2_PO_4_, pH 7.2–7.4) as described elsewhere [47]. Briefly, grounded aliquots (0.5 g) were weighed and added to a centrifuge tube (50 mL) and extracted (×2) with 5 mL of PBS at 37 °C with gentle agitation for 2 h. Afterwards, the suspension was centrifuged for 15 min at 2000× *g*. Total soluble protein concentrations in the extracts was determined using a Lowry method-based commercial kit (TP0200, Sigma, St. Louis, MO, USA).

### 4.4. Cell Culture Conditions

The human hepatoblastoma (HepG2) HB-8965^®^ cell line was obtained from the American Type Culture Collection (Rockville, MD, USA) at passage 1 and used in experiments between passages 4–8. The cells were grown in Eagle’s Minimum Essential Medium (EMEM Glutamax, Gibco, Life Technologies, Madrid, Spain) supplemented with 10% fetal bovine serum (Gibco) and Hepes, 1% (Sigma). The cells were maintained at 37 °C in 5% CO2, 95% air, and the culture medium was changed every 2 days. For the experiments, cells were seeded at (1) a density of 105 cells/well in 24-well plates (Costar, Cambridge, MA, USA), which were used for lysosomal activity and AhR ligand analyses, or (2) a density of 106 cells/well in 12-well plates (Costar, Cambridge, MA, USA), which were used for mRNA expression analyses. The cell cultures were grown with 1 mL of the EMEM. At 24 h post seeding, the cell cultures were challenged (3 h) with 5% (*v*/*v*) of the raspberry polyphenolic extract (0.5 nmol/L, PP treatment), pooled plasma samples from eight Wistar rats fed with a high-fat diet (HF treatment) or a standard diet (AIN-93G) (ST treatment), protein extract obtained from *Triticum durum* (50 µg of proteins, TD treatment) and a combination of the HF and TD with PP treatment. 

### 4.5. Lysosomal Activity

The activity was investigated using the neutral red (toluylene red; 3-amino-7 dimethylamino-2-methylphenazine hydrochloride) uptake assay described in a previous study [48]. Culture medium was spent out and cells were washed twice with PBS. The uptake of toluylene red was measured using a commercial kit (Sigma, nr 7H092), and absorbance was measured at 540 nm with background subtraction at 690 nm.

### 4.6. Real-Time Reverse Transcription-Polymerase Chain Reaction (RT-qPCR)

Total RNA was extracted from cell cultures with TRI Reagent^®^ Solution (Cat No. AM9738, Invitrogen^TM^) according to the manufacturer’s instructions. Total RNA (0.45 µg) was converted to double-stranded cDNA using AMV Reverse Transcriptase (Cat No. M9004 Promega, Madison, WI, USA). PCR was performed with primers designed (www.ncbi.nlm.nih.gov) for the following genes (Homo sapiens): toll-like receptor 4 (TLR4, forward 5′-TAC TGC ACA AGG TGA GGT GTT-3′, reverse 5′-TGT CTC AGC CAA CTG CCT AC-3′), aryl hydrocarbon receptor (AhR, forward 5′-TCG ATG TAT CAG TGC CAG CC-3′, reverse 5′-ATG CTG TCT CCA TGA ATG CTG T-3′), cyclooxygenase-2 (COX-2, forward 5’-ATT CCG CTG CAA GAA GAC GA-3′, reverse 5′-AGA GAA TGA TTT A TTT GCT GTC CTT-3′), angiopoietin-like 4 (ANGPTL4) (forward 5′-GCA TGG CTG CCT GTG GTA AC-3′, 5′-ATC TTG CTG TTT TGA GCC TTG A-3′ reverse), oxidized low-density lipoprotein receptor (oxLDL, forward 5′-AAA GGA CCC CTA GAG TCG CA-3′, reverse 5′-ACA GAC AGG CTC CAA GGA ATG-3′), nucleotide-binding oligomerization domain 2 (NOD2, forward 5′- GCC TTC CTT CTA CAG CAC GT-3′, reverse 5′-TGG CAG GGC TCT TCT GCA AG -3′), signal transducer and activator of transcription 1 (STAT1, forward 5′-GCA GGT TCA CCA GCT TTA TGA-3′, reverse 5′-TGA AGA TTA CGC TTG CTT TTC CT-3′), cluster of differentiation 44 (CD44, forward 5′-ACC AAG AAG ACA TCG ATG CC-3′, reverse 5′-TGT CCA GCT AAT TCG GAT CC-3′) and glyceraldehyde 3-phosphate dehydrogenase (GAPDH, forward 5′-CCA CTC CTC CAC CTT TGA CG-3′; reverse 5′-CGC CAG ACC CTG CAC TTT TT-3′). The latter was used as a housekeeping gene. The PCR mix (10 µL reaction volume) consisted of 5 µL SYBR™ Select Master Mix (Applied Biosystems, Woolston, UK), 1 µmol/L primers, and 2 µL cDNA. The PCR reactions were performed in triplicate in a LightCycler (Applied Biosystems) system using the following conditions: 1 cycle at 95 °C for 5 min and 35 cycles at 60 °C for 20 s and 72 °C for 45 s. The mRNA expression of the tested gene relative to GAPDH expression was calculated using the 2^−ΔCp^ method. Samples of each cell culture were measured in duplicate, and gene expression was expressed as fold-change.

### 4.7. Analysis of the AhR Ligand

Hepatocytes were fixed with an ethanol:PBS (60:40, *v*/*v*) solution for 10 min at room temperature. Cells were permeabilized with 0.05% Triton X-100 for 10 min and treated with the anti-AhR (1:50) (BD Biosciences, Franklin Lakes, NJ, USA) antibody overnight at 4 °C. Cells were imaged with an inverted fluorescence microscope (Leica DM IL LED Fluo, Wetzlar, Germany). Arbitrary units of the AhR ligand in hepatocytes were determined using ImageJ software (version 1.43 n). 

### 4.8. Statistical Analyses

Statistical analysis was performed using Statistica software, version 10.0 (StatSoft Corp., Kraków, Poland). Data were analyzed using a one-way ANOVA, followed by Duncan’s post hoc test. Data from plasma lipid profiles were analyzed using a Student’s *t* test. Statistical significance was established at *p* ≤ 0.05.

## 5. Conclusions

The study assessed the effects of different obesogenic-related markers modulated by raspberry polyphenolic extract in hepatocytes and their related associations. The raspberry polyphenolic extract reduced neutral red uptake, and thus might modulate intracellular (i.e., absorption and vesicle trafficking) responses. Exposure of HepG2 cells to the examined polyphenolic extract favorably influenced the expression levels of immunometabolic mediators activated by the HF and TD treatments. Moreover, the kinetic analysis of AhR ligand expression showed that HF-related molecular mechanisms activated AhR expression earlier than the examined extract started the regulatory effect in the hepatocytes. The observed dynamism between PP and HF in AhR expression might be useful in potential further dietary recommendations to regulate molecular signals activated by a high-fat diet.

## Figures and Tables

**Table 1:** Polyphenol composition of the raspberry extract.

Compound	Mean (*n* = 3)	SD
Ellagitannins (ET) and ellagic acid conjugates (EAC)		
Sanguiin H-10 iso.1	607.6	5.4
Lambertianin C-EA	117.0	1.6
Sanguiin H-10 iso.2	545.6	5.1
Lambertianin C	979.9	18.5
Sanguiin H-6	5403.8	29.6
Ellagic acid	354.7	1.5
Total ET	7653.8	56.4
Total EAC	760.2	3.9
Total ET + EAC	8414.0	60.2
Anthocyanins (ACY)		
Cy-spoh	4435.5	28.9
Cy-glu-rut	359.8	0.6
Cy-glu	1345.6	12.1
Cy-rut	142.6	0.2
Plg-glu	58.6	2.2
Total ACY	6342.1	40.5
Flavanols (FLAVA)		
(+)-catechin	22.9	0.7
(−)-epicatechin	358.3	1.0
Proanthocyanidins	602.9	3.8
mDP	2.2	0.0
Terminal units [%]	45.5	0.2
(+)-catechin	4.1	0.4
(−)-epicatechin	41.4	0.5
Extension units [%]	54.5	0.2
(+)-catechin	45.2	0.3
(−)-epicatechin	9.2	0.3
Total FLAVA	984.1	2.3
Total phenolic content (TPH)		
TPH	15,740.2	25.6

Values are expressed as the mean ± standard deviation (mg/100 g); *n*—number of measurements; Cy-spoh—cyanidin-3-spohoroside; Cy-glu-rut—cyanidin-3-glucosyl-rutinoside; Cy-glu—cyanidin-3-glucoside; Cy-rut—cyanidin-3-rutinoside; Plg-glu—pelargonidin-3-glucoside; mDP—mean degree of polymerization; TPH—total polyphenols; terminal and extension units represent percent of the (+)-catechin and (−)-epicatechin placed in the terminal or extension position of flavanols chemical structure.

## Supplementary Materials

The following are available online.
