# Supplementary material for: Raspberry Polyphenolic Extract Regulates Obesogenic Signals in Hepatocytes
Source: Molecules. 2018 Aug 21;23(9):2103. doi: 10.3390/molecules23092103 (PMC6225237; doi:10.3390/molecules23092103)
Supplement: Supplementary file 1 [file molecules-23-02103-s001.pdf]

# Raspberry Polyphenolic Extract Regulates Obesogenic Signals in Hepatocytes

**Bartosz Fotschki [1,*], José Moisés Laparra [2] and Michał Sójka [3]**

## Supplementary Material 1

Production of the juice

Clear raspberry juices were produced at processing line in the Institute of Horticulture in Skierniewice from the frozen raspberries cv. ‘Polana’. Fruits were purchased from the commercial cold store, thawed, enzyme treated and pressed. Juice was centrifuged and pasteurised.

The processing line equipped with steam pots with a water jacket (ZE.6, Lozamet, Łódź, Poland) where fruits were thawed and heated up to 50°C. Then fruits were disintegrated with a grinding mill with a perforated disc with a mesh diameter of 6 mm (BASIS 91/55, Fryma-Maschinen AG, Rheinfelden, Germany). Hot mash was depectinised with the Rohapect 10 l (AB Enzymes, Darmstadt, Germany) at a dose of 200 g/1000kg, at 50°C until a negative result of the pectin test, but for a period not shorter than one hour. The pulp was pressed with a Bucher rack-and-cloth press. Obtained raw juice was centrifuged using a continuous-flow disc-stack centrifuge (LAB 102B-25, Alfa Laval, Brentford, Middlesex, Great Britain) at 1500 rpm (125 × g). Clear juice was then hot-filled into 5 l bags after heating with a continuous flow pasteurizer (P20-VB, Alfa-Laval Food Engineering, Lund, Sweden) at a temperature above 92°C and a flow rate that allowed the juice to stay in the heat exchanger for 30 seconds. After pasteurisation, the juice was cooled in individual containers in running water and stored in the cold room at 2°C until delivery for further processing.
